# Tick-borne encephalitis virus induces chemokine RANTES expression via activation of IRF-3 pathway

**DOI:** 10.1186/s12974-016-0665-9

**Published:** 2016-08-30

**Authors:** Xiaowei Zhang, Zhenhua Zheng, Xijuan Liu, Bo Shu, Panyong Mao, Bingke Bai, Qinxue Hu, Minhua Luo, Xiaohe Ma, Zongqiang Cui, Hanzhong Wang

**Affiliations:** 1 Key Laboratory of Special Pathogens and Biosafety, Center for Emerging Infectious Diseases, Wuhan Institute of Virology, Chinese Academy of Sciences, Xiaohongshan No.44, Wuhan, 430071 China; 2 State Key Laboratory of Virology, Wuhan Institute of Virology, Chinese Academy of Sciences, Xiaohongshan No.44, Wuhan, 430071 China; 3 Beijing 302 Hospital, Beijing, 100039 China; 4 CAS Center for Excellence in Brain Science and Intelligence Technology (CEBSIT), Shanghai, China

**Keywords:** Tick-borne encephalitis virus, Neuroinflammation, Chemokine, RANTES, IRF-3

## Abstract

**Background:**

Tick-borne encephalitis virus (TBEV) is one of the most important flaviviruses that targets the central nervous system (CNS) and causes encephalitides in humans. Although neuroinflammatory mechanisms may contribute to brain tissue destruction, the induction pathways and potential roles of specific chemokines in TBEV-mediated neurological disease are poorly understood.

**Methods:**

BALB/c mice were intracerebrally injected with TBEV, followed by evaluation of chemokine and cytokine profiles using protein array analysis. The virus-infected mice were treated with the CC chemokine antagonist Met-RANTES or anti-RANTES mAb to determine the role of RANTES in affecting TBEV-induced neurological disease. The underlying signaling mechanisms were delineated using RANTES promoter luciferase reporter assay, siRNA-mediated knockdown, and pharmacological inhibitors in human brain-derived cell culture models.

**Results:**

In a mouse model, pathological features including marked inflammatory cell infiltrates were observed in brain sections, which correlated with a robust up-regulation of RANTES within the brain but not in peripheral tissues and sera. Antagonizing RANTES within CNS extended the survival of mice and reduced accumulation of infiltrating cells in the brain after TBEV infection. Through in vitro studies, we show that virus infection up-regulated RANTES production at both mRNA and protein levels in human brain-derived cell lines and primary progenitor-derived astrocytes. Furthermore, IRF-3 pathway appeared to be essential for TBEV-induced RANTES production. Site mutation of an IRF-3-binding motif abrogated the RANTES promoter activity in virus-infected brain cells. Moreover, IRF-3 was activated upon TBEV infection as evidenced by phosphorylation of TBK1 and IRF-3, while blockade of IRF-3 activation drastically reduced virus-induced RANTES expression.

**Conclusions:**

Our findings together provide insights into the molecular mechanism underlying RANTES production induced by TBEV, highlighting its potential importance in the process of neuroinflammatory responses to TBEV infection.

**Electronic supplementary material:**

The online version of this article (doi:10.1186/s12974-016-0665-9) contains supplementary material, which is available to authorized users.

## Background

Tick-borne encephalitis (TBE), an endemic in many regions of Europe and Asia, is an important emerging infectious disease that targets the central nervous system (CNS) caused by the TBE virus (TBEV; family Flaviviridae, genus Flavivirus). TBEV consists of three subtypes: the European subtype (TBEV-Eu) in most parts of Europe; Siberian subtype in eastern Europe, Russia, and northern Asia; and Far Eastern subtype (TBEV-FE) in eastern Russia and some parts of China and Japan. TBEV-Eu is mainly transmitted by *Ixodes ricinus* and the other two subtypes by *Ixodes persulcatus* [1]. In humans, TBEV causes a variety of clinical manifestations, ranging from flu-like febrile disease to encephalitis of differing severity levels [2]. The clinical outcome may in part depend upon the subtype of TBEV infection. TBEV-Eu and TBEV-Sib subtypes are usually associated with milder disease, with mortality rates of 0.5–2 %. In contrast, infection with the TBEV-FE subtype results in the most severe CNS disorder, with mortality rates of up to 40 % and higher rates of severe neurologic sequelae [3, 4].

The first TBEV replication usually occurs in dendritic cells of the skin following tick bites, later in regional lymph nodes, and then virus can be detected in plasma [5, 6]. During the stage of active viremia, virus may cross the blood-brain barrier (BBB) and invade the CNS where it causes profound destruction of nerve cells [2]. The most severe forms of TBE may be characterized by major damage to neurons in different parts of the brain and spinal cord [4]. Generally, CNS pathology is the consequence of viral infection of corresponding cells and the resulting neuroinflammatory responses. In clinical studies, common findings include immunohistochemical staining of TBEV antigen in large neurons of human brains of fatal cases with relatively short natural clinical course. However, topographical correlation between inflammatory changes and distribution of viral antigens is poor, since affected regions with prominent inflammatory infiltrates and marked neuronal damage contained only few immunolabeled structures [7]. Furthermore, it was found that granzyme B-releasing cytotoxic T cells contribute significantly to neuronal damage in human TBE [8], supporting the notion that liberation of inflammatory mediators and recruitment of cytotoxic T cells may contribute to nerve cell dysfunction in human TBEV infection. In a TBEV-infected mouse model, CD8^+^ T cells was also shown to play a pivotal role in the immunopathology of TBE as evidenced by prolonged survival of severe combined immunodeficiency (SCID) or CD8^−/−^ mice following infection, compared with immunocompetent mice or mice with adoptively transferred CD8^+^ T cells [9].

These results imply that immunopathological effects significantly contribute to the onset of TBE. However, the exact mechanisms of proinflammatory effects responsible for immune-mediated neuronal injury are still unclear with limited data available on the role of chemoattractant cytokines (chemokines) during TBEV infection. Although proinflammatory chemokines C-X-C motif chemokine 10 (CXCL10), C-X-C motif chemokine 11 (CXCL11), monocyte chemoattractant protein-1 (MCP-1), and regulated upon activation, normal T cell expressed, and presumably secreted (RANTES) have been detected in the cerebrospinal fluid (CSF) samples of TBE patients [10–14], the specific impact on inflammatory responses and the molecular mechanisms that regulate chemokine expression remain to be further addressed.

Chemokines constitute a family of small, secreted proteins that orchestrate leukocyte migration to sites of inflammation, playing a crucial role in the regulations of homeostasis by trafficking specific cells under physiologic conditions [15, 16]. However, overproduction of chemokines in response to immunologic, inflammatory, infectious signals may elicit deleterious effects, especially in the largely non-self-renewing brain tissues [17]. Therefore, a clearer understanding of how chemokines impact the inflammatory response to viral infections within the CNS is important for identifying targets that can potentially be manipulated for the development of host defense with minimal negative effects. To date, there is limited information concerning specific chemokines responsible for cell recruitment and their potential impact on disease progression during TBEV infection.

In the present study, we have evaluated chemokine expression profiles within CNS after TBEV infection using mouse as a model. We demonstrate that TBEV induces marked inflammatory cell infiltrates in the brain. RANTES has been shown to be one of the main chemokines induced within CNS during primary TBEV infection [12, 14]. Blockade of RANTES reduces the accumulation of infiltrating cells and extends the survival of mice after TBEV infection. Furthermore, our data indicate that stimulation of interferon regulatory factor 3 (IRF-3) pathway leads to RANTES secretion by TBEV-infected human brain-derived cells. Together, our results identify RANTES as a potential mediator of the neuroinflammatory responses seen in TBEV infection, providing basic insights into the molecular mechanism underlying TBEV-induced RANTES production.

## Methods

### Ethics statement

All experimental procedures were conducted according to the guidance of the Institutional Animal Care and Use Committee of Wuhan Institute of Virology, Chinese Academy of Sciences. All surgeries were performed under sodium pentobarbital anesthesia, and all efforts were made to minimize suffering as well as the number of animals used.

### Viruses and cell cultures

WH2012, a Far Eastern strain of TBEV, was isolated from tick (*I. persulcatus*) samples and characterized as previously described [18]. The nucleotide acid sequence of WH2012 strain was deposited in the GenBank database (accession number KJ755186). Working stocks of TBEV were routinely propagated on Vero cells. All procedures with infectious materials were performed under biosafety level-3 (BSL-3) conditions.

BHK-21, Vero, SK-N-SH (a human neuroblastoma cell line; ATCC HTB-11), and T98G (a human glioblastoma cell line; ATCC CRL-1690) cells were cultured in Minimum Essential Medium (MEM). CCF-STTG1 (a human astrocytoma cell line; ATCC CRL-1718) and THP-1 (a human monocytic cell line; ATCC TIB-202) were grown in RPMI 1640 medium. A549 (a human alveolar epithelial cell line, ATCC CCL-185) cells were cultured in F-12K medium. All media were supplemented with 10 % fetal bovine serum, 2 mM l-glutamine, 100 U ml^−1^ penicillin and 100 mg ml^−1^ streptomycin (Life Technologies, NY). Human progenitor-derived astrocytes (HPDAs) were generated from neural progenitor cells and cultured as previously described [19, 20]. Differentiation of THP-1 (2 × 10^5^ cells/ml) monocytes into macrophage-like cells was achieved using 200 nM phorbol 12-myristate 13-acetate (PMA; Sigma, NY) for 3 days as previously described [21].

### Mice infection and monitoring

Groups of 1-week-old BALB/c mice of either sex were challenged with TBEV WH2012 by intraperitoneal (ip) or intracerebral (ic) inoculation route with a volume of 20 μl of virus suspensions at various concentrations, using Hamilton syringe (Hamilton Co., Switzerland). Mock-infected animals were inoculated with 20 μl of diluent (serum-free DMEM). Groups of female mice aged 7–8 weeks were infected intracerebrally with 10^3^ median tissue culture infective dose (TCID_50_)s of TBEV WH2012. Mice were then monitored daily for signs of neurological disease and survival over a period of 12 to 25 days. Mortality rate was assessed at the time points indicated below.

### Tissue isolation and preparation

Cohorts of virus-infected mice were sacrificed after ic inoculation with a virus dose containing 10^3^ TCID_50_s for the time points indicated below. Whole blood was obtained by cardiac puncture, and sera were separated by centrifugation. Following perfusion with ice-cold phosphate-buffered saline (PBS), the small intestine, hind limb muscle, heart, lung, spleen, liver, and brain were collected, homogenized, and cleared by low-speed centrifugation. Then, sera and tissue samples were immediately stored at −80 °C for further analysis.

### Virus titration

Virus infectivity was determined by estimation of the TCID_50_ using standard cell culture conditions. Briefly, BHK-21 cells were seeded in 96-well plates. When the cells reach 80 % confluence, they were infected with 150 μl of serial decimal dilutions of each sample for 4 days. The cytopathic effect was thereby monitored by microscopic examination, and the infectivity titer was expressed as TCID_50_/g tissue using the Reed-Muench formula.

### Histology

After anesthesia with sodium pentobarbital (60 mg kg^−1^), mice were transcardially perfused with ice-cold PBS followed by 4 % (*w*/*v*) paraformaldehyde (PFA) in PBS. The brains were dissected and post-fixed overnight, then trimmed, and routinely paraffin wax embedded. Serial 3–5-μm-thick sections were stained with hematoxylin-eosin (HE) for histological analysis under a light microscopy.

### Protein array analysis

Brains of mice were screened for 40 inflammatory factors using a commercialized mouse inflammation antibody (Ab) array C1 Kit according to the manufacturer’s instructions (RayBiotech, Inc., Norcross, GA). Briefly, brain sections were pooled (*n* = 4/group), homogenized in lysis buffer (kit component), and then centrifuged at 12,000 rpm for 15 min at 4 °C. The membranes were incubated with the supernatants at a final concentration of 1 μg/μl. Chemiluminescent blot documentation was performed with a FluorChem HD2 imaging system.

### In vitro TBEV infection

T98G, CCF-STTG1, SK-N-SH, HPDAs, A549, and differentiated THP-1 cells were infected with TBEV WH2012 at a multiplicity of infection (MOI) of 1. To assess the requirement for viral replication in the generation of RANTES, cells were treated with UV-inactivated preparations of TBEV. At the indicated time points, culture supernatants and cell monolayers were harvested for further analysis.

### RANTES mRNA quantification

Total RNA was extracted from cell monolayers using Omega HP total RNA Isolation kit (Omega Bio-Tek, Inc., GA). To remove residual genomic DNA, RNA samples were pretreated with RQ1 RNase-free DNase (Promega, WI). Then, RNA was converted to cDNA using M-MLV Reverse Transcriptase (Promega). Real-time PCR was carried out using primers specific for human RANTES (sense 5′-ACCACACCCTGCTGCTTTGC-3′, antisense 5′-CCGAACCCATTTCTTCTCTGG-3′) and TransStart Eco Green qPCR SuperMix kit (TransGen, Beijing, China) on the Bio-Rad CFX96 Real-Time PCR System (Bio-Rad Laboratories, Inc., CA). The data acquisition and analysis were carried out with CFX Manager Software (version 2.1; Bio-Rad). Relative expression of RANTES was calculated using the 2^−ΔΔCt^ method [22] after normalization with endogenous control β-actin (sense 5′-CGGGAAATCGTGCGTGACAT-3′, antisense 5′-GAACTTTGGGGGATGCTCGC-3′). Results are expressed as the relative fold increase of the stimulated over the mock control group.

### RANTES ELISA

The mouse tissue homogenates and cell culture supernatants were assessed for RANTES protein levels using RANTES-specific enzyme-linked immunosorbent assay (ELISA) kit (Boster, Wuhan, China), according to the manufacturer’s instructions. The analytical procedure has been described previously [20].

### Chemotaxis

Cell chemotaxis assay was performed as previously described [23, 24]. Briefly, THP-1 cells (5 × 10^5^ cells/100 μl) were added to the upper chamber of Transwell insert (5-μm polycarbonate filter; Corning, NY). A total of 1-ml culture supernatant from TBEV-infected T98G (MOI = 5) for 72 h was added to the lower chamber of Transwell insert. For the neutralizing experiment, anti-hRANTES Ab (0.5 μg/ml; R&D Systems, Minneapolis, MN) or control normal goat IgG was added to the conditioned media in the lower chamber. Plates were incubated for 4 h at 37 °C and 5 % CO2. Transwell inserts were then removed, and the number of cells migrated to the lower chamber was determined with a TC20 Automated Cell Counter (Bio-Rad). Data are expressed as percentage of the migrated cells in total number of input cells.

### Anti-RANTES treatment

Cohorts of mice were treated via ic injection with Met-RANTES (1 μg/mouse; Bachem, Basel, Switzerland), vehicle, anti-RANTES mAb (MAB478), or an IgG2a isotype-matched control mAb (10 μg/mouse; R&D Systems) on days 2 to 8 after ic infection with TBEV WH2012 (10^3^ TCID_50_s). Met-RANTES is a recombinant RANTES analog, in which the initiating methionine residue is retained after expression in *Escherichia coli* cells, resulting in a potent antagonist of the murine RANTES receptors CC chemokine receptor (CCR)5 and CCR1 [25, 26]. The chosen RANTES-neutralizing mAb reacts with murine and human RANTES and no other identified cytokine or chemokine [27, 28]. Mice were monitored daily for signs of neurological disease and survival over a period of 14 days. The survival curves were compared using Kaplan-Meier tests. Both brain virus titers and mice survival rates were estimated at the time points indicated below. The severity of inflammation was determined by staining sections of paraffin-embedded brain tissue with HE by day 8 post infection (p.i.), as after this time, the efficacy of this treatment may decline due to the decay of corresponding molecules within the mouse brain.

### Plasmids and cell transfection

The RANTES promoter reporter construct (pGL2-220) and site-mutated plasmids for cAMP response element (CRE) (CRE Mut), interferon-stimulated response element (ISRE) (ISRE Mut), nuclear factor for interleukin 6 (NF-IL6) (NF-IL6 Mut), nuclear factor kappa-light-chain-enhancer of activated B cells (NF-kB1) (NF-kB1 Mut), and NF-kB2 (NF-kB2 Mut) were kindly given by Dr. Casola (University of Texas Medical Branch, USA) [29]. The dominant-negative mutants of IkB kinase alpha (mIkBα), IRF-3ΔN, and IRF-7ΔN were kind gifts of Prof. S. B. Xiao (Huazhong Agricultural University, China). All plasmids for transfection were prepared with Endo-free Plasmid Midi Kit (Omega Bio-Tek, Inc., GA). Exponentially growing T98G cells in 24-well plates were transfected with 0.1 μg of pRL-TK reporter (Renilla luciferase for internal control) and 0.5 μg of RANTES-pGL2 plasmids (Firefly luciferase, experimental reporter) using the X-tremeGENE HP reagent (Roche, Mannheim, Germany). For co-expression experiments, 0.5 μg of each indicated expression plasmid was added to the reporter vectors. Twenty-four hours after transfection, cells were inoculated with TBEV and harvested at the indicated time interval. Reporter gene activity was measured using a dual-luciferase assay system (Promega, Madison, WI), according to the manufacturer’s instructions.

### Western blot analysis

T98G cells were washed with PBS and lysed with RIPA lysis buffer (Beyotime, Nantong, China) containing protease inhibitor (cOmplete Protease Inhibitor Cocktail Tablets; Roche). Equal amounts of protein were separated on 12 % SDS-PAGE and transferred onto an Immobilon-P PVDF membrane (Millipore, MA). The blots were blocked for 1 h with 2 % bovine serum albumin in Tris-buffered saline (20 mM Tris-HCl, pH 7.4, 0.15 M NaCl) containing 0.1 % Tween 20 (TBST) at room temperature (RT) and reacted overnight at 4 °C with the primary antibodies. The membranes were washed four times with TBST and then incubated with the secondary Ab for 1 h at RT. Horseradish peroxidase-conjugated goat anti-rabbit IgG (#31460) or goat anti-mouse IgG (#31430) secondary antibodies were used (Thermo Scientific Pierce, IL). The membranes were washed and visualized with BeyoECL Plus Chemiluminescent Substrate (Beyotime) for signal detection. Primary antibodies directed against p-IRF-3 (Ser396, 4D4G, #4947), p-TANK-binding kinase 1 (TBK1) (Ser172, D52C2, #5483), retinoic acid-inducible gene 1 (RIG-I) (D14G6, #3743), melanoma differentiation-associated protein 5 (MDA5) (D74E4, #5321), IkBα (#9242), and TBK1 (#3013) were obtained from Cell Signaling Technologies (Beverly, MA). IRF-3 (#11312-1-AP) and β-actin (#60008-1-Ig) antibodies were purchased from Proteintech Group, Inc. (Chicago, IL).

### RNA interference

T98G cells were transfected with small interfering RNA (siRNA) duplexes using HiPerFect transfection reagent (Qiagen, Hilden, Germany) according to the manufacturer’s protocols. siRNA oligonucleotides were synthesized (RiboBio, Guangzhou, China) with the following sequences (sense strands): RIG-I, 5′-GGAAGAGGUGCAGUAUAUU-3′; MDA5, 5′-GGUGAAGGAGCAGAUUCAG-3′ [30]. Nonsilencing siRNA with a scrambled sequence were used as a negative control (control siRNA (Ctrl siRNA)). After cultured for 16 h, the medium with transfection reagent was removed. Cells were either mock treated or infected with TBEV at an MOI of 1 for 24 h. Culture supernatants and cell monolayers were harvested for further analysis.

### Inhibition of cellular signaling pathway

Inhibition of IRF-3 and NF-kB signaling in T98G and CCF-STTG1 cells was performed with BX795 (InvivoGen, CA) and MG132 (Millipore), respectively, as described previously [31, 32]. Briefly, virus inoculation were carried out as mentioned above, followed by treatment with BX795 (2 μM), MG132 (3 μM), or DMSO vehicle in the absence of serum for 36 h, and the levels of RANTES released in culture supernatants were determined by ELISA. IRF-3 and p-IRF-3 were detected by Western blot analysis. Virus infectivity was determined by TCID_50_ assay as described above.

### Statistical analyses

Data were expressed as means ± SD. All statistical analyses were carried out using SigmaPlot®10.0 software (Stystat Software, CA), with a *P* value of <0.05 considered statistically significant.

## Results

### Virulence studies

**Fig. 1:**
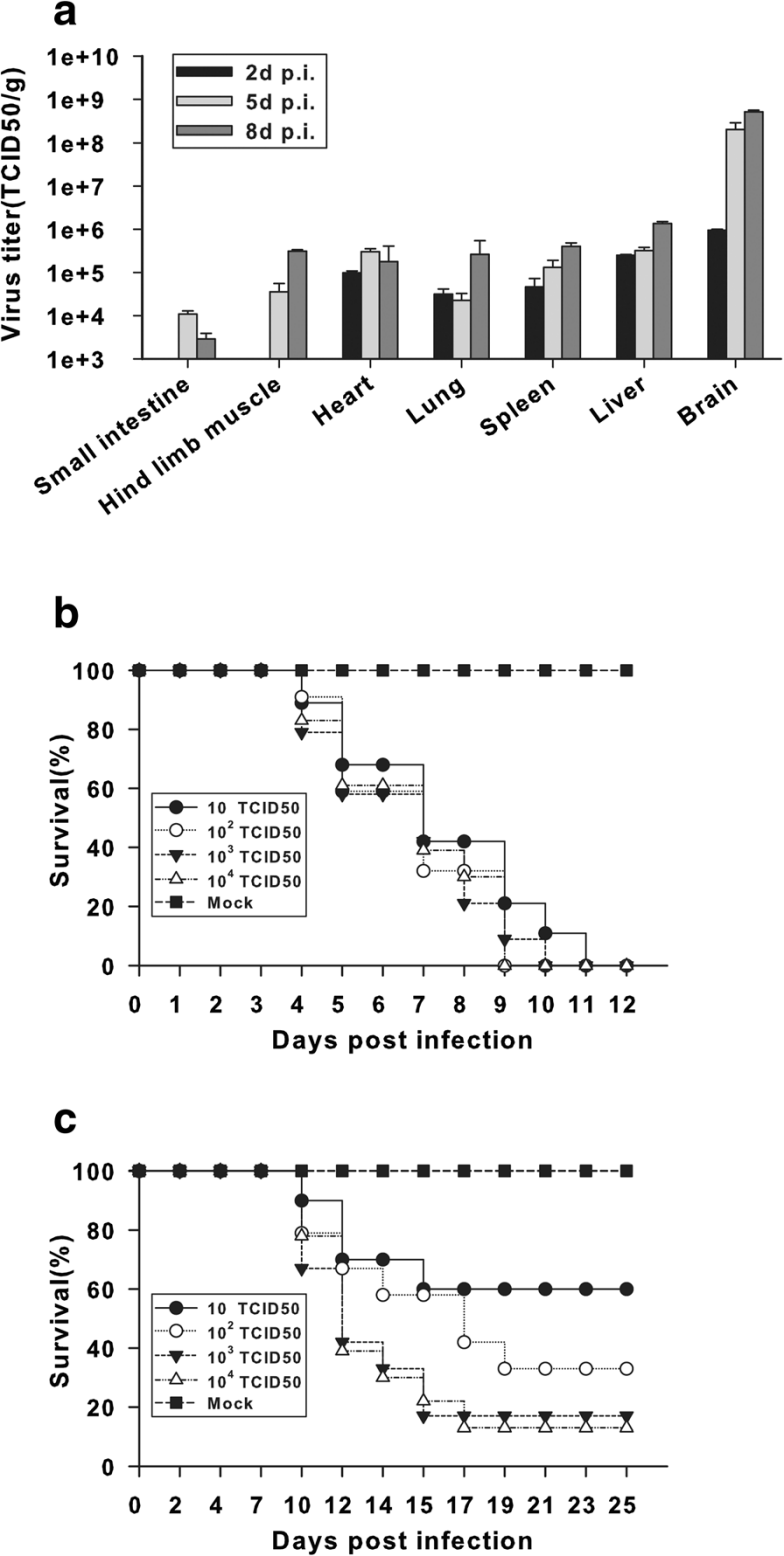
Morbidity and mortality of mice infected with TBEV. **a** TBEV burden in different tissues by ic route in mice. Litters of BALB/c mice were challenged with TBEV WH2012 (10^3^ TCID_50_s) by ic route. The kinetics of virus replication and levels of TBEV were determined in selected tissues of virus-infected mice at indicated time points (days 2, 5, and 8) by TCID_50_ assay. For each time point, the virus titers are the average of four mice. Bars represent the means ± the standard deviations of three independent experiments (*n* = 4 mice per group). **b** Survival rates following ic inoculation with serial challenge doses of TBEV. Survival curves were recorded for 12 days. Data shown are pooled from three independent experiments (total of approximately 30 mice per group). **c** Survival rates following ip inoculation with serial challenge doses of TBEV. Survival curves were recorded for 25 days. Data shown are pooled from three independent experiments (total of approximately 30 mice per group)

After ic inoculation of 1-week-old mice with TBEV (10^3^ TCID_50_s), most animals developed neurological symptoms, such as hunched posture, loss of balance, hind limb paralysis, and convulsions in the final stage of the disease prior to death (data not shown). Major organs were harvested at various time points to determine virus titers in mice infected with TBEV. Virus was initially detected between 2 and 5 days p.i., with virus titers peaking on day 8 p.i. except for small intestine and heart. With respect to viral distribution, significantly higher virus titers were recovered from the brain, but relatively mediocre levels of viruses were also detected in peripheral tissues (Fig. 1a). Following ic infection of mice with sequentially increasing doses, all tested groups exhibited fatal outcomes, with no significant differences in the mean survival times between the challenge doses (Fig. 1b). However, following ip (Fig. 1c), subcutaneous (sc) [18] or intramuscular (im) (data not shown) infection of groups of mice with sequentially increasing doses, 100 % mortality was not observed even with the highest tested virus challenge dose. These observations indicate that direct CNS infection induces early death in mice even after low-dose challenge.

### TBEV induces RANTES within host CNS

**Fig. 2:**
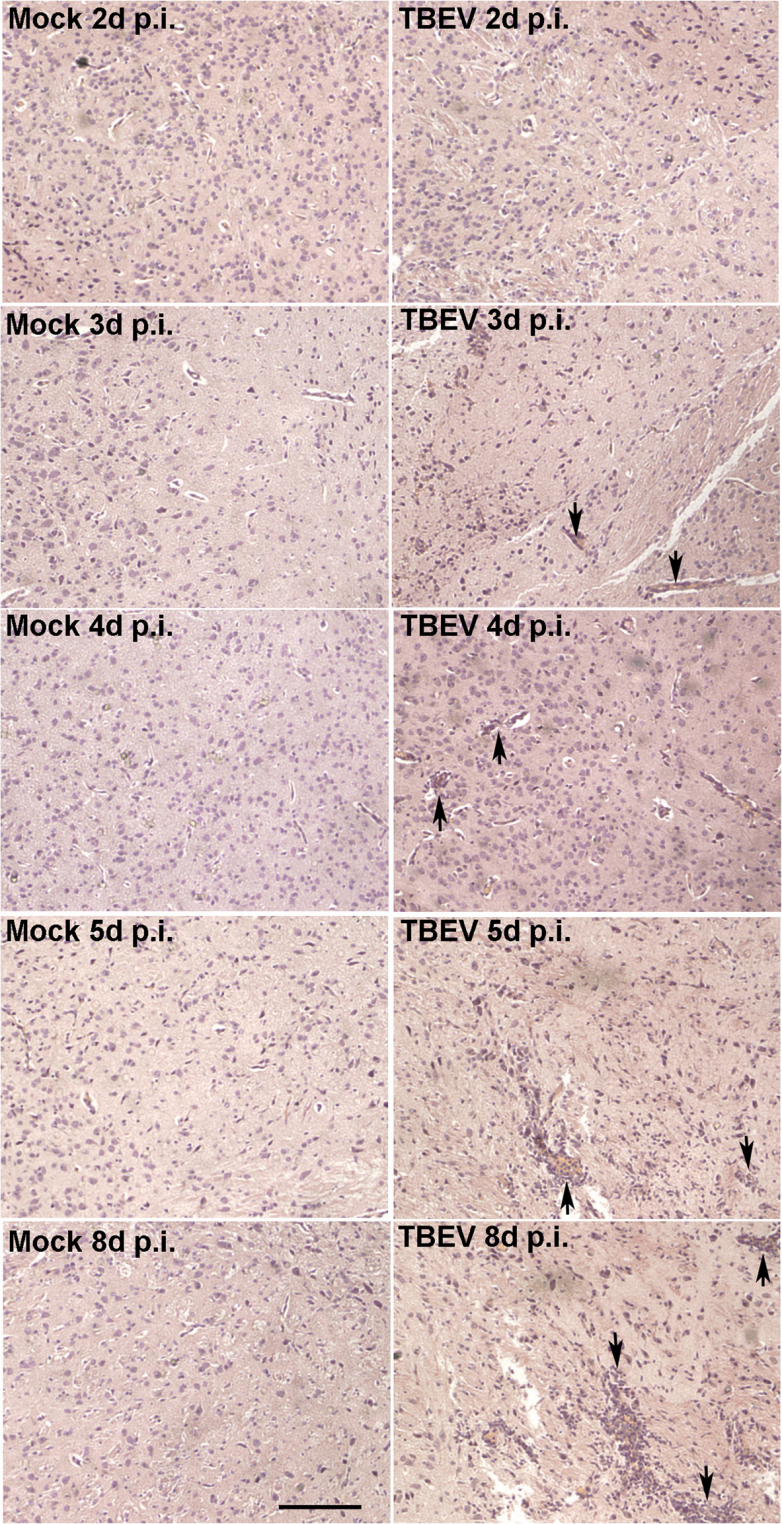
Histopathological analyses for the TBEV-infected mice brain. The histopathological changes of PFA-fixed sections in cerebral cortex were examined by HE staining at the denoted time points after TBEV or mock infection. *Black arrows* identify leukocytes infiltrating into the brain tissues. The photomicrographs demonstrate representative images obtained from three independent experiments (*n* = 4 mice per group). *Bars*, 100 μm

To gain insight into the neuroinflammatory responses elicited specifically in the CNS compartment, TBEV WH2012 strain was inoculated by the ic route. Time course of acute brain injury was assessed histologically. As depicted in Fig. 2, the HE-stained brain sections of the cerebral cortex obtained from virus-infected group indicate inflammatory cell infiltrates appeared as early as 3 days post challenge. By day 4 p.i., the inflammatory reaction increased. Marked inflammatory cell infiltrates were observed on day 8 p.i. when nearly all infected mice succumbed to infection.

**Fig. 3:**
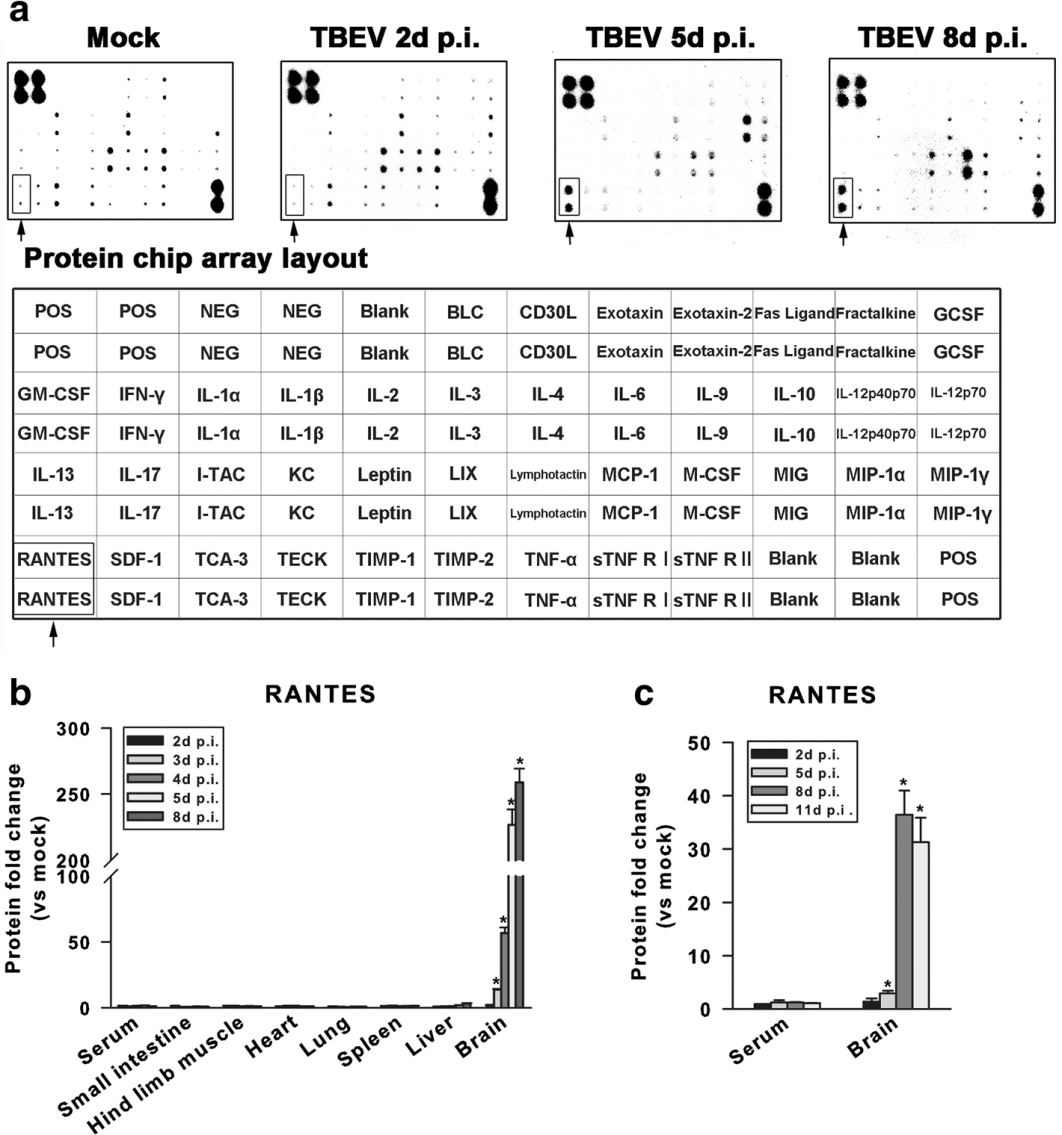
TBEV infection induces RANTES production within the brain of mice. **a** Expression of 40 inflammatory factors in the mice brain detected by an inflammation Ab array assay. Representative photomicrographs demonstrate that TBEV infection stimulated RANTES (indicated by *arrows*) expression. Data shown are pooled from three independent experiments (*n* = 4 mice per group). **b** Time course of TBEV-induced stimulation of RANTES protein expression in different tissues. *Bars* represent the means ± the standard deviations of three independent experiments (*n* = 4 mice per group). The fold changes in expression of RANTES in the brain of TBEV-infected mice were significantly higher when compared to those of the peripheral tissues and sera on days 3, 4, 5, and 8 p.i. (^*^ *P* < 0.05). **c** Fold changes of RANTES protein levels in the brain and serum of TBEV-infected adult mice. Bars represent the means ± the standard deviations of three independent experiments (*n* = 4 mice per group). The fold changes in expression of RANTES in the brain of TBEV-infected mice were significantly higher when compared to that of the sera on days 5, 8, and 11 p.i. (^*^ *P* < 0.05)

CNS infiltration of peripheral immune cells requires antecedent local expression of cytokines, chemokines, and cell adhesion molecules [33]. To identify the key immune factors induced by TBEV in CNS, brains of virus-infected mice were harvested on days 2, 5, and 8 p.i., and the expression of 40 inflammatory factors was profiled by a mouse-specific protein array. Compared with baseline expression in uninfected controls, the secretion of a panel of immune factors was increased in mouse brains after virus inoculation. Among them, the CC-chemokine RANTES was induced within 5 days after TBEV infection, and the expression levels also remained elevated until the moribund state (Fig. 3a). Below, the expression of RANTES upon TBEV infection is characterized.

To confirm the protein array data, induction of RANTES was examined by ELISA. Mediocre induction of RANTES in response to TBEV infection was found as early as 3 days post challenge (13.9 ± 0.6-fold increase). Then, sustained up-regulation of RANTES in CNS was observed from day 4 to day 8 after infection. Closely mimicking the results from the protein array analysis, a robust increase in RANTES protein expression was observed on day 5 (226.6 ± 11.7-fold increase) and persisted up to day 8 (258.8 ± 10.5-fold increase) p.i. within the brain tissue. Interestingly, the induction pattern of RANTES expression appeared to be coincided with the dynamics of immune cell infiltration in the CNS. It is noteworthy that no concomitant increase of RANTES expression was recorded in tested peripheral tissues and sera (Fig. 3b). Besides, the protein abundance for RANTES (in pg/ml) in the brain was also greater than those observed in tested peripheral tissues and sera following ic TBEV infection (data not shown). To strengthen that the results obtained from 1-week-old mice are also valid in adult mice, RANTES protein levels in the brain and serum were also measured in mice aged 7–8 weeks after ic infection with TBEV. By day 5 p.i., a slight increase in RANTES expression occurred in the brain of adult mice (2.9 ± 0.5-fold increase). TBEV infection caused a dramatic increase in the level of brain RANTES on days 8 (36.4 ± 4.5-fold increase) or 11 (31.3 ± 4.6-fold increase) p.i. compared to mock controls (Fig. 3c).  There was no obvious induction of RANTES production in the serum, and RANTES level in the brain of TBEV-infected adult mice was also significantly higher when compared to that in the serum (data not shown). This higher RANTES concentration in the brain of virus-infected adult mice as compared to that in the serum suggest potential localized inflammatory responses. Taken together, these observations suggest that TBEV induces a significant increase of RANTES production within CNS, which is likely related to the progression of neuroinflammatory responses.

### Antagonizing RANTES slows down CNS disease progression in TBEV-infected mice

**Fig. 4:**
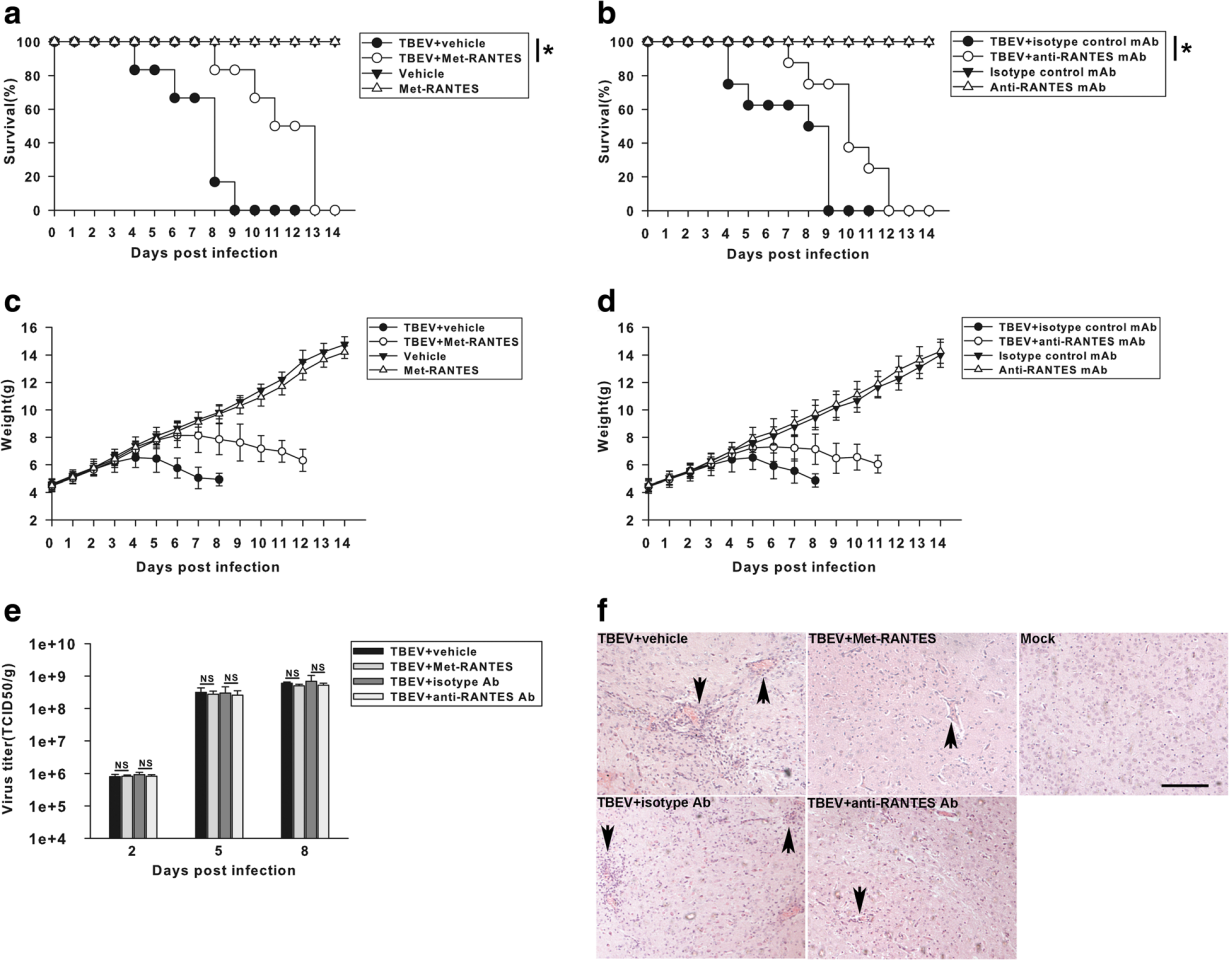
Antagonizing RANTES within CNS extends the survival of mice after TBEV infection. Mice were treated with Met-RANTES, vehicle, anti-RANTES mAb, or isotype-matched control Ab daily from day 2 to 8 after a primary TBEV infection. **a** Met-RANTES-treated mice exhibited a delay in mortality following lethal TBEV challenge. Mortality in each group was monitored daily for 14 days. Data were pooled from three independent experiments (total of approximately 18 mice per group). Statistical differences were evaluated using the Kaplan–Meier test for mortality. *P < 0.05, compared to vehicle-treated mice. **b** Anti-RANTES mAb-treated mice exhibited a delay in mortality following lethal TBEV challenge. Mortality in each group was monitored daily for 14 days. Data were pooled from three independent experiments (total of approximately 18 mice per group). *P < 0.05, compared to isotype Ab-treated mice. **c**, **d** Body weight changes of mice were monitored daily. For each time point, the measured values are the average of the surviving mice. *Bars* represent the means ± the standard deviations of three independent experiments (total of approximately 18 mice per group). **e** Infectious virus in brain tissues. No significant difference in virus titers was observed at day 2, 5, or 8 between Met-RANTES-treated mice and vehicle-injected mice or anti-RANTES mAb-treated and isotype Ab-injected mice. *Bars* represent the means ± the standard deviations of three independent experiments (*n* = 4 mice per group). *NS* not significant. **f** Met-RANTES or anti-RANTES mAb treatment reduced inflammatory cell accumulation (indicated by *black arrows*) in cerebral cortex sections, compared to results for vehicle- or isotype Ab-treated mice, respectively. The histopathological changes of PFA-fixed sections in cerebral cortex were examined by HE staining on day 8 p.i. The photomicrographs demonstrate representative images obtained from three independent experiments (*n* = 4 mice per group). *Bars*, 100 μm

Previous studies have demonstrated that TBEV induced significant alterations in brain physiology that appeared to closely parallel the pattern of RANTES expression. To determine whether antagonizing the activity of this chemokine within CNS would affect disease progression, virus-infected mice were treated with either Met-RANTES or anti-RANTES mAb. As shown in Fig. 4a, b, all infected vehicle, Met-RANTES, isotype control mAb, and anti-RANTES mAb-treated mice eventually succumbed to TBEV infection. However, both Met-RANTES and anti-RANTES mAb-treated mice showed a significant delay in mean time to death after infection, compared with corresponding control groups. The mean survival time for isotype control mAb-treated infected mice was approximately 7.1 days, while anti-RANTES mAb-treated mice succumbed to mortality later after infection (mean days until death, 10.0 days). TBEV-infected Met-RANTES-treated mice lived an average of approximately 4 days longer (mean days until death, 11.3), compared with virus-infected vehicle-treated mice (mean days until death, 7.2). Moreover, TBEV-infected mice treated with Met-RANTES (Fig. 4c) or anti-RANTES mAb (Fig. 4d) exhibited delayed growth retardation, compared with virus-infected vehicle- or isotype Ab-treated animals, respectively. The intensity of symptoms increased gradually up to a moribund state, at a much slower rate for TBEV-infected mice treated with Met-RANTES or anti-RANTES mAb, compared with vehicle- or isotype Ab-injected animals, respectively (data not shown). These results suggest that antagonizing RANTES within CNS affects disease progression following lethal ic challenge with TBEV.

In an effort to identify the potential contributing factors to the enhanced survival of anti-RANTES-treated mice, we assess virus replication in the brain of infected mice. As shown in Fig. 4e, no significant difference was observed in TBEV titer recovered from the brain of Met-RANTES-treated mice compared with that in vehicle-injected mice. On day 2, 5, and 8 p.i., virus titers in brain tissue were also similar between the infected anti-RANTES mAb-treated and isotype Ab-treated groups. Morphologically, treatment of TBEV-infected mice with either RANTES receptor antagonist Met-RANTES or anti-RANTES mAb resulted in a significant decrease in local infiltration of inflammatory cells. In addition, less severe perivascular cuffing and hemorrhage within brains after Met-RANTES or anti-RANTES mAb administration, compared to the pathological manifestations present within the cerebral cortex of vehicle or isotype Ab-treated mice was found (Fig. 4f). Therefore, it is likely that blockade of RANTES within CNS reduced accumulation of infiltrating cells and extended the survival of mice after TBEV infection. Overall, our data highlight a potential role for RANTES within CNS in affecting neuroinflammation during TBEV infection.

### TBEV up-regulates RANTES production in human brain-derived cells

**Fig. 5:**
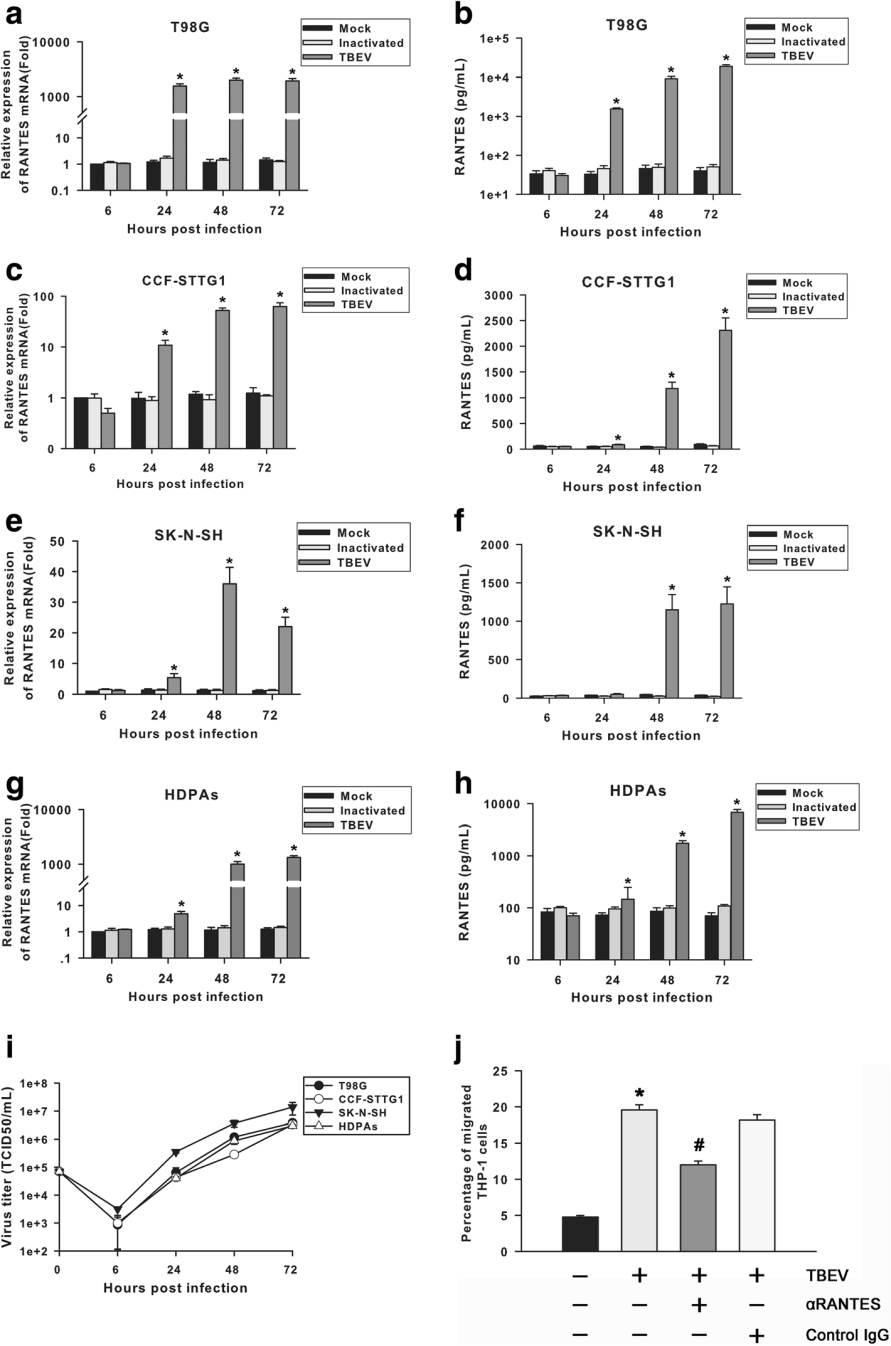
RANTES is up-regulated in TBEV-infected human brain-derived cells. T98G (**a**, **b**), CCF-STTG1 (**c**, **d**), and SK-N-SH (**e**, **f**) cells and HPDAs (**g** and **h**) were inoculated with medium alone, TBEV, or UV-inactivated TBEV at an MOI of 1. Total RNA was extracted from cell lysates at 6, 24, 48, and 72 h post inoculation. RANTES mRNA was quantified by real-time PCR (**a**, **c**, **e**, and **g**), and results were normalized to GAPDH and expressed as fold induction over medium alone at 6 h post inoculation. Supernatants were harvested at 6, 24, 48, and 72 h post inoculation, and levels of RANTES (pg/mL) released were determined by ELISA (**b**, **d**, **f**, and **h**). *Bars* represent the means ± the standard deviations of three independent experiments. *P < 0.05 versus mock control. **i** TBEV replication kinetics in human brain cells. T98G, CCF-STTG1, and SK-N-SH cells and HPDAs were infected with TBEV at an MOI of 1. Supernatants were collected at indicated time points, and virus titers were determined by TCID_50_ assay. The results are presented as the means ± standard deviations obtained from three independent experiments. **j** Neutralization of RANTES in T98G cell supernatants with specific anti-hRANTES Ab (αRANTES) reduced THP-1 cell migration. The same amount of normal goat IgG was used as controls. Data are expressed as the percentages of migrated cells in total cells added and presented as means ± the standard deviations of three independent experiments. *P < 0.05 versus control; ^#^ *P* < 0.05 versus TBEV-infected alone. Statistical analysis was performed by Student’s *t* test

The primary target of TBEV is the brain. To investigate the mechanism by which TBEV induces RANTES in the CNS, the ability of TBEV infection to trigger RANTES expression in human brain-derived cell lines was assessed. TBEV significantly induced RANTES production both at the messenger RNA (mRNA) and protein levels in human glioblastoma cells T98G at 24 h p.i. and reached a high level at 72 h p.i. compared with mock-infected controls (Fig. 5a, b). Human astrocytoma cells CCF-STTG1 and neuroblastoma cells SK-N-SH were also capable of significantly expressing RANTES in response to TBEV infection, but the level of RANTES expression was generally much lower than that in T98G cells (Fig. 5c–f). To examine whether TBEV infection induces RANTES expression in primary CNS cells, further experiments were conducted using HPDAs as a model. RANTES transcription was induced by TBEV infection, with the increase started at 24 h p.i. and peaked at 72 h p.i. (Fig. 5g). The protein levels of RANTES reached the peak at 72 h p.i., and the pattern was similar to that of the three cell lines described above (Fig. 5h). In tested virus-infected brain cells, the up-regulation of RANTES gene expression by TBEV was likely replication dependent, since UV inactivation dramatically impaired the ability of TBEV to induce RANTES mRNA expression and protein secretion. To reveal the kinetics of virus replication, virus titers were measured for the indicated time points by TCID_50_ assay. As shown in Fig. 5i, TBEV replicated efficiently in all cell lines tested. Increasing titers of infectious virus were observed after 24 h p.i., and the maximum titer of virus was attained after 72 h p.i. Given the above-described RANTES expression profile, its production appeared to be significantly induced at time points that coincided with significant virus replication in human brain-derived cells. It was also found that viral replication was coupled to RANTES up-regulation in alveolar epithelial A549 cells (Additional file 1: Figure S1A–C). However, though TBEV could replicate in human monocytic cell line THP-1, RANTES production was not significantly induced after infection of this cell line, demonstrating a cell type-specific pattern of RANTES production (Additional file 1: Figure S1D–F). To conclude, the human brain cells are capable of significantly producing RANTES upon TBEV infection, although the level of induced RANTES may vary depending on the cell type.

We further determined the chemotactic activity of the secreted RANTES from TBEV-infected T98G cells using a transwell migration assay. Supernatants from these cultures were more potent in chemoattracting THP-1 cells, compared with those from uninfected control cells. Notably, the chemoattractant activities of these conditioned medium were largely reduced when incubated with anti-hRANTES Ab, suggesting that RANTES is a functionally active chemotactic component secreted by TBEV-infected T98G cells (Fig. 5j).

### TBEV-induced RANTES expression is mediated through transactivation of RANTES promoter

**Fig. 6:**
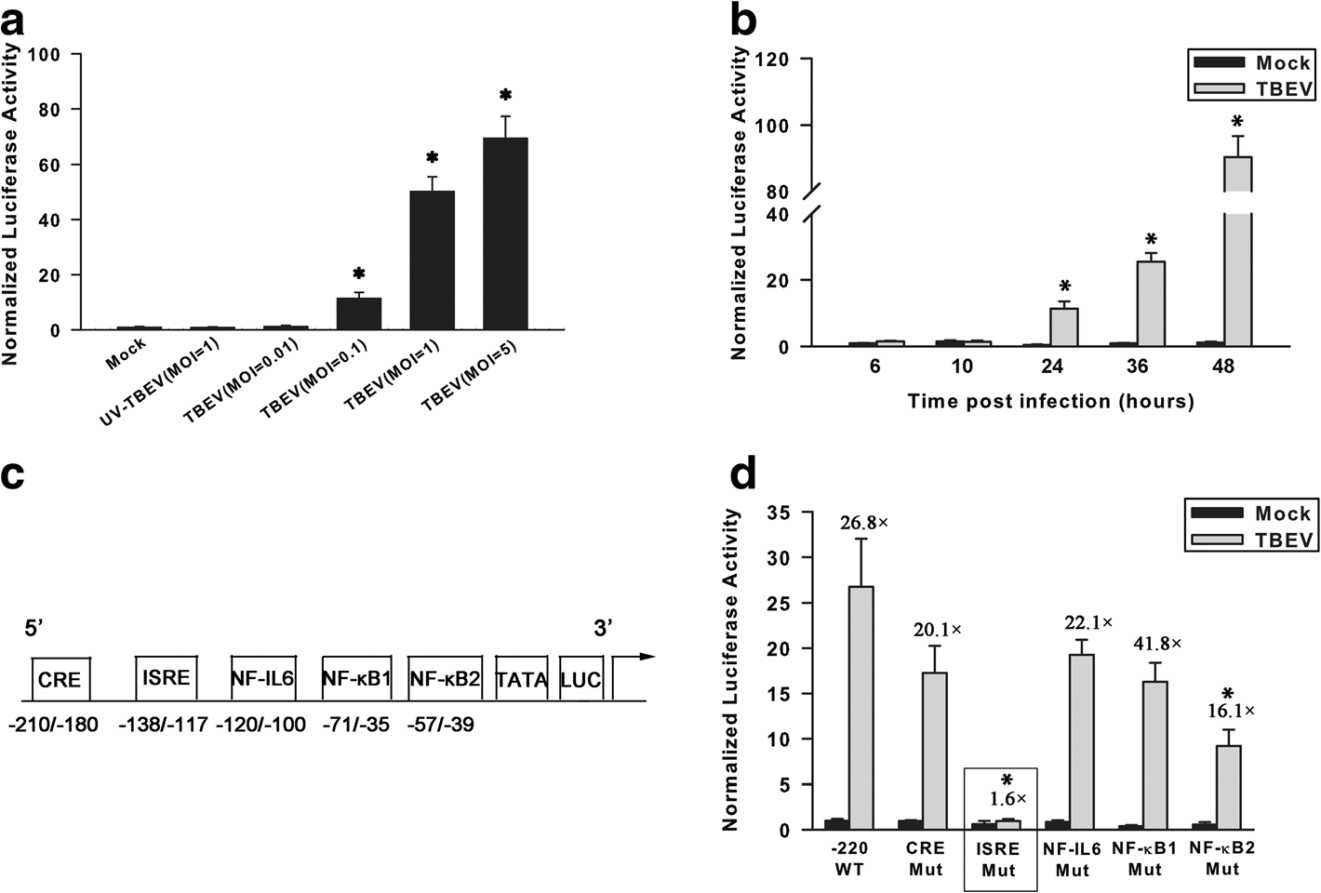
TBEV infection results in the induction of RANTES promoter activity. **a** T98G cells were co-transfected with pGL2-220 and pRL-TK, followed by UV-inactivated TBEV or TBEV infection at different MOIs; 24 h p.i. cells were harvested to measure luciferase activity. The results are expressed as fold induction of RANTES promoter activity relative to the basal level. *P < 0.05 versus mock control. **b** T98G cells were co-transfected with pGL2-220 and pRL-TK, followed by TBEV infection at an MOI of 0.1. At indicated times p.i., cells were harvested to measure luciferase activity. The results are expressed as fold induction of RANTES promoter activity relative to the basal level. *P < 0.05 versus mock control. **c** Schematic representation of the RANTES promoter constructs [29]. Locations of the putative binding sites for CRE, ISRE, NF-IL-6, and NF-kB are illustrated. Numbering is relative to the transcription initiation site. **d** Effect of site mutations in the RANTES promoter sequence on TBEV-inducible activity. T98G cells were transiently transfected with site-mutated (Mut) plasmids of the pGL2-220 RANTES promoter and infected with TBEV for 36 h. The results are expressed as fold induction of RANTES promoter activity relative to the basal level. *Bars* represent the means ± the standard deviations of three independent experiments. *P < 0.05 compared with pGL2-220 plus TBEV infection

To determine whether TBEV infection transactivates the RANTES promoter, a reporter plasmid pGL-220 which contains the sequence from nucleotides −220 to −55 (relative to the mRNA start site) was co-transfected with pRL-TK into T98G cells, followed by TBEV infection. T98G was chosen as a cell model due to its relatively high transfection efficiency and the ability of the cells to produce high levels of RANTES transcripts upon TBEV infection. As shown in Fig. 6a, TBEV infection of transfected T98G cells induced a dose-dependent increase of luciferase activity, with the maximal stimulation detected at an MOI of 5. UV-inactivated virus was not capable of inducing RANTES transcription, confirming our previous observation that RANTES induction requires productive TBEV infection. The TBEV-induced promoter activation was also time-dependent, with the increase of luciferase activity started at 24 h and reached the highest levels at 48 h p.i. (Fig. 6b).

The RANTES promoter comprises five pivotal binding sites for the transcription factors CRE, ISRE, NF–IL-6, NF-kB1, and NF-kB2 (Fig. 6c) [29]. To determine the contributions of individual *cis* regulatory elements in conferring responsiveness to TBEV infection, we tested the impact of mutations at these sites on TBEV-induced RANTES production. As shown in Fig. 6d, mutation of the ISRE site not only affected the promoter basal activity but also nearly completely abolished TBEV-induced RANTES promoter activation. Mutation of the NF-kB1 or NF-kB2 site also reduced the basal activity, with the latter decreasing the TBEV-induced luciferase activity, although to a much lesser extent. Taken together, these data indicate that ISRE element of RANTES promoter is possibly mainly responsible for TBEV-induced RANTES expression.

### TBEV-induced RANTES expression is mediated by activation of IRF-3 signaling pathway

To clarify the essential role of ISRE, the effects of ISRE-binding IRF-3 and IRF-7 transcription factors lacking their DNA-binding domains (mutants IRF-3ΔN and IRF-7ΔN, respectively) on TBEV-induced RANTES expression were determined. In reporter gene assays, compared with corresponding controls, over-expression of IRF-3ΔN dramatically inhibited the ability of TBEV in activating RANTES gene transcription, whereas co-transfection with IRF-7ΔN in TBEV-infected T98G cells did not have such impact (Fig. 6a). Moreover, over-expression of a nondegradable IkBα mutant (mIkBα) had no effect on activation of the RANTES promoter (Fig. 6b).

To determine whether TBEV trigger RANTES expression by directly inducing activation of known IRF-3 pathway component, we performed Western blot analysis to detect the levels of endogenous RIG-I, MDA5, TLR3, p-TBK-1, p-inhibitor-kB kinase epsilon (IKKε), and p-IRF-3 following infection of T98G cells with TBEV. As depicted in Fig. 6c, TBEV induced up-regulation of RIG-I and MDA5 as early as by 24 h p.i., whereas no detectable TLR3 and p-IKKε were seen during the process of infection in T98G cells (data not shown). Concomitantly, virus replication also activates specific virus-induced kinase TBK-1, which regulates the phosphorylation and thus the activation of IRF-3 [34]. Indeed, phosphorylated forms of IRF-3 (p-IRF-3) were clearly detectable by 24 h p.i. However, we failed to detect a significant decrease in the level of endogenous IkBα, which is a hallmark of IkBα degradation and NF-kB activation.

**Fig. 7:**
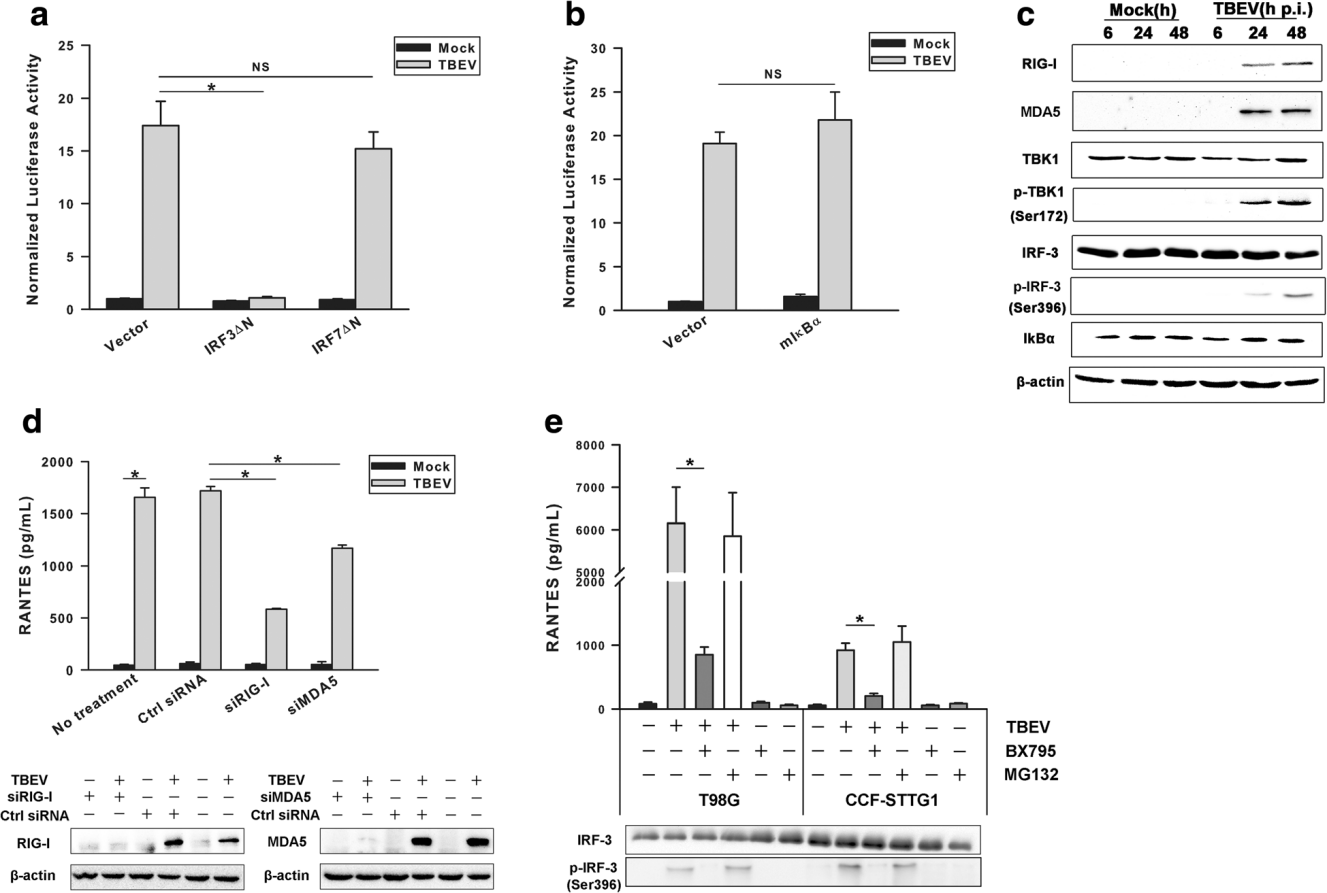
TBEV-induced RANTES expression is mediated by activation of IRF-3 pathway. **a** T98G cells were co-transfected with pGL2-220 and pRL-TK together with an empty vector or a dominant-negative mutant IRF-3 (IRF-3ΔN) or IRF-7 (IRF-7ΔN) and infected or uninfected with TBEV at an MOI of 1. Cells were harvested at 24 h post infection to measure luciferase activity. The results are expressed as fold induction of RANTES promoter activity relative to the basal level. *Bars* represent the means ± the standard deviations of three independent experiments. **b** The experiments were performed similar to those described in **a** except that a dominant-negative mutant IkBα plasmid (mIkBα) was used. *Bars* represent the means ± the standard deviations of three independent experiments. **c** Western blot analysis of phosphorylation state of IRF-3 in TBEV-infected cells. Whole-cell lysates were recovered from mock- and TBEV-infected T98G cells over a 48 h time course, and Western blot analysis was performed to examine levels of p-IRF-3, IRF-3, p-TBK1, TBK1, RIG-I, MDA5, IkBα, and β-actin. Three independent experiments were performed, and one representative result was shown. **d** Effect of RIG-I or MDA5 siRNA on RANTES expression in TBEV-infected cells. T98G cells were transfected with indicated siRNA. After 16-h incubation, the cells were mock-infected or TBEV-infected (MOI = 1) for 24 h. Supernatants were harvested and analyzed for RANTES protein expression (pg/ml) by ELISA (*upper panel*). *Bars* represent the means ± the standard deviations of three independent experiments. Expression of RIG-I or MDA5 proteins was assessed by Western blot analysis (*lower panel*). β-actin serves as a loading control. One representative result of three is shown. **e** T98G and CCF-STTG1 cells were infected with TBEV at an MOI of 1, followed by treatment with BX795, MG132, or DMSO vehicle in the absence of serum for 36 h. Levels of RANTES (pg/ml) released were determined by ELISA (*upper panel*). *Bars* represent the means ± the standard deviations of three independent experiments. Western blot was performed to examine levels of p-IRF-3 or IRF-3 (*lower panel*). One representative result of three is shown. *P < 0.05 indicates significant difference between groups tested. *NS* not significant

The abovementioned results indicate that the expression of both RIG-I and MDA5 is greatly enhanced in response to TBEV infection. To explore the role of these proteins in modulation of virus-induced RANTES production, we performed knockdown of RIG-I and MDA5 using siRNA. As shown in Fig. 7d, Ctrl siRNA did not affect the virus-induced activation. Knockdown of either RIG-I or MDA5 led to a significant decrease in the amount of RANTES chemokine production under the experimental conditions. These results indicate the involvement of endogenous RIG-I and MDA5 in the up-regulation of RANTES upon TBEV infection in T98G cells.

To confirm the actual contribution of TBK-1/IRF-3 module to the TBEV-induced RANTES expression, T98G cells were treated with BX795, an inhibitor blocking TBK1- and IKKε-mediated activation of IRF-3, followed by TBEV infection. BX795 significantly blocked the TBEV-induced phosphorylation of IRF-3 at Ser396. Furthermore, secretion of RANTES from T98G and CCF-STTG1 cells was significantly blocked in the presence of BX795. In contrast, treatment with MG132, a potent inhibitor of NF-kB activation, had no effect on RANTES release in virus-infected cells. Therefore, it appears that TBEV-induced production of RANTES is unlikely dependent on the NF-kB signaling pathway (Fig. 7e). It should be noted that both BX795 and MG132 did not significantly block virus replication under the conditions of the experiment (Additional file 2: Figure S2). These results suggest that activation of IRF-3 signaling pathway is most likely to be involved in the TBEV-induced RANTES expression.

## Discussion

To focus our study on specific immune mechanisms involved in CNS response to viral infection rather than on aspects of extracerebral infection and neuroinvasion, we challenged mice with TBEV via ic route. Pathological analysis of CNS tissues from moribund mice demonstrated marked leukocytes infiltration, which is consistent with autopsy studies on human patients infected with TBEV [7, 8]. Once in the CNS, there are three possible mechanisms by which flaviviruses induce brain tissue destruction leading to the clinical manifestations of disease. Virus infection may directly lead to neuronal cell injury and virus-induced neuroinflammatory responses may cause neuronal death, or both [9, 35]. CNS pathology of TBEV is considered to be, at least in part, due to viral infection of corresponding cells, since virus induces both apoptosis and necrosis in human neural cells and also in mouse and monkey brain neurons [36–39]. However, there is a growing body of evidence indicating that abnormal immune response is one major cause of tissue damage and fatal encephalitis [40]. TBEV infection of the CNS has been shown to result in markedly enhanced leukocyte migration into the brain tissue and immune-mediated BBB breakdown, both of which corresponded with excessive expression of chemokines and cytokines in the brain parenchyma [41]. Using an Ab array that detects 40 immune factors, we observed that TBEV infection stimulated several inflammatory mediators, including chemokine RANTES and MCP-1 and cytokines IL-12p40p70, IL-12p70, and IL-4. Our findings are in line with previous studies on chemokine and cytokine profiles of mouse CNS infected with the Neudoerfl strain of TBEV-Eu subtype or the Sofjin strain of TBEV-FE subtype, demonstrating a common induction pattern of these immune mediators among TBEV infection [41, 42]. It is noteworthy that we did not observe a significant increase of certain proinflammatory cytokines including tumor necrosis factor (TNF)-α and IL-6. This could be due to the sensitivity of the protein array adopted, various immune responses of distinct mouse models, or the difference in inducing specific cytokines by different TBEV strains. These need to be clarified in future studies.

During TBEV infection, the expression of proinflammatory molecules may contribute to the influx of peripheral lymphocytes in the brain as well as to the severity of the encephalitis [7, 8, 14]. In this report, we found that CC chemokine RANTES was one of the most rapidly and rigorously induced molecule in the CNS during TBEV infection. The increasing level of RANTES expression between 3 and 8 days p.i. was shown to be consistent with increasing immune cell infiltrates and neuroinflammation in virus-infected 1-week-old mice. Besides, ic infection of TBEV in adult mice also induced a significant increase in the level of RANTES within CNS, but not in sera. These findings suggest that the capability of inducing RANTES expression by TBEV infection seems not to depend on age of the mouse. Even though the neonatal immune system is somewhat less mature than those of adult, neonates are not immune privileged, especially under high inflammatory conditions. A large body of evidence indicated that the mouse neonatal immune system is capable of mounting virus-specific T cell-based immune responses, as well as protective memory and Ab responses [43–45]. In other studies, increased expression of RANTES was found in neonatal mice after infection with viruses such as coxsackievirus B3 [46] and influenza virus [47] or protozoan parasite such as *Cryptosporidium parvum* [48]. It should be noted that though neonatal mice are a relatively high-sensitive model for the study of flavivirus infections, peripheral administration of WH2012 strain via either ip, sc [18], or im (data not shown) route did not lead to 100 % lethality. These data indicate that WH2012 strain used in this study is less pathogenic, as compared with some highly virulent TBEV isolates, such as strain Hypr [49] and strain Sofjin [50].

RANTES is usually significantly induced following viral infection, and its production represents a characteristic of neuroinflammation [51]. It has been reported that several viruses including human immunodeficiency virus type 1 (HIV-1), herpes simplex virus-1 (HSV-1), Japanese encephalitis virus (JEV), mouse hepatitis virus (MHV), and rabies virus (RABV) can up-regulate RANTES production within CNS [52–56]. Moreover, we demonstrated a TBEV-induced robust expression of RANTES in human brain-derived cell cultures that recapitulated the cell types normally found in the brain including neurons, astrocytes, and microglia. It is worth noting that RANTES was dramatically increased neither in tested peripheral tissues nor in sera. Similar findings were previously reported in human cases of TBE, showing that RANTES was significantly increased in CSF, but not in sera [12, 14]. Furthermore, our in vitro culture systems showed that TBEV induces the production of RANTES which is functionally active in recruiting human monocytic cells. Therefore, the strikingly high expression of RANTES within CNS is likely to be one of the mediators that form a concentration gradient in the brain during TBEV infection. For describing a specific involvement of RANTES in experimental TBE, future research should define the activated cell types which will be recruited into CNS after RANTES production upon TBEV infection.

In this work, we observed that treatment of mice with Met-RANTES or anti-RANTES mAb prolonged survival and decreased cellular infiltrates in the brain. In agreement with our study, a previous report showed that, in a severe herpes simplex encephalitis mouse model, treatment with either Met-RANTES or anti-RANTES mAb decreased leukocyte recruitment into the brain of HSV-1-infected mice [54]. Moreover, Met-RANTES treatment significantly reduced proinflammatory chemokine or cytokine production in the CNS and prolonged survival time of the mice after RABV infection [56]. Considering the important role of RANTES in exerting a potent chemotactic effect on both monocytes and T cells, our data suggest that both immune cells infiltrate reduction and survival extension in treated TBEV-infected mice was, at least in part, correlated with the blockade of RANTES alone. Early studies demonstrated the importance of CCR5, which is one of the receptors of RANTES, as a protective factor in the context of flaviviral infections. With the use of knockout mice, it was demonstrated that CCR5 deficiency reduced immune cell infiltration of the CNS and increased mortality after peripheral inoculation of WNV [57] or JEV [58]. In humans, homozygosity for the CCR5Δ32 allele is associated with the predisposition to the clinical TBE in Lithuanian people [59, 60], but not Russian people [61]. These findings suggest that CCR5 deficiency is probably relevant to a weakened immune defense against evading flaviviruses. However, it can be speculated that direct administration of a receptor antagonist to the brain is clearly different to a systemic loss of the receptor, which may be ascribes to significant difference between the immune response elicited in the CNS and the response in the periphery. Moreover, it would be expected that antagonizing RANTES alone may differ from blockade of CCR5, in which both binding of a number of chemokines and recruitment of main immune cell types may be affected. In this study, we were not able to draw any conclusions on relationships between disease severities of TBE and a functional RANTES-CCR5 axis. Future studies with conditional *RANTES* and *CCR5* knockout mouse models may help delineating the pathogenic mechanisms of the disease.

It is worth noting that despite remarkable prolongation of survival times of infected mice after Met-RANTES and anti-RANTES mAb treatments, both strategies led to no change in viral burdens following a high inoculum of TBEV injection in the brain. Therefore, it seems that blockade of RANTES within CNS appears to result in alteration of the immune/inflammatory response, rather than a simple modulation of increase or decrease in the level of TBEV infection. Since both viral infection and host immune responses likely contribute to the pathogenesis of TBE, enhancement of the antiviral activity against TBEV and amelioration of the neuroinflammatory response may theoretically help in reducing the severity of the disease. Further studies are needed to determine whether concomitant administration of antivirus drugs together with anti-inflammatory agents could offer an additive beneficial effect on TBE. With respect to other chemokines induced by TBEV, antagonizing MCP-1 within CNS did not significantly ameliorate TBEV infection (data not shown). However, future research is warranted to explore whether some of the detected or as yet unidentified immune mediators contribute individually and synergistically to neuroinflammatory responses in the process of TBEV infection.

In this study, we found that TBEV infection could induce RANTES production in human brain-derived cell lines and primary progenitor-derived astrocytes in vitro. This up-regulation was not detectable as early as 6 h p.i., and measurable RANTES mRNA expression and protein release only occurred between 24 and 48 h p.i. The results observed here are reminiscent of some previous studies on delay of IFN induction upon TBEV infection. At early stages of infection, viral double-stranded RNA (dsRNA) was mainly found within ER-derived vesicles, thus to be largely unavailable for cytoplasmic pattern recognition receptors (PRR) [62, 63]. Similarly, the mechanism behind the delay of RANTES production might also be that TBEV induces replication vesicles, thereby delaying the detection of viral dsRNA by PRR sensors. Although it is known that TBEV infection can induce RANTES production in vivo and in vitro, the underlying mechanism contributing to the induction of RANTES after TBEV infection has not been explored. Using brain-derived cells as a model, we demonstrated that TBEV infection activated the RANTES promoter in both time- and dose-dependent manners. It is generally believed that expression of many of the chemokine genes is regulated primarily at the level of transcription, and their promoter regions contain recognition sites for virus-activated transcription factors [64]. Binding sites for a wide variety of transcription factors annotated within the *RANTES* promoter include IRFs, NF-kB, CCAAT/enhancer-binding protein (C/EBP), and cAMP response element-binding protein (CREB)/activating protein 1 (AP-1) [29, 65]. These transcription factors have been shown to contribute to the differential regulation of *RANTES* gene expression, depending on the virus and the cell type [53, 66–69]. We revealed in this study that TBEV-induced transcription of RANTES is mainly mediated by activation of the IRF-3 pathway. This conclusion is based on several lines of evidence: (i) mutation of the ISRE site almost completely abolished TBEV-induced promoter activation; (ii) over-expression of IRF-3, but not IRF-7 or IkBα dominant-negative mutant, efficiently inhibited TBEV-induced RANTES production; (iii) TBEV infection triggered the phosphorylation of endogenous IRF-3 in a time-dependent manner; and (iv) addition of inhibitor targeting TBK1–IRF-3 signaling pathway considerably reduced RANTES production in T98G and CCF-CTTG1 cells.

Flavivirus infections produce virus replicative intermediate dsRNA, which could be detected by the cytoplasmic RNA sensor RIG-I and MDA5 [70, 71]. By interacting with mitochondrial adapter protein mitochondrial antiviral signaling protein (MAVS), RIG-I/MDA5 directs the activation of TBK1 and IKKε [72–74]. Activated TBK1 mediates IRF-3 phosphorylation, which ultimately leads to the transcription of type I IFN and other cellular genes with host defense functions. For instance, phosphorylation of IRF-3 can directly stimulate RANTES transcription [66]. In other scenarios, IRF-3 is also of particular importance in synergistically promoting RANTES expression, together with NF-kB activation [67, 75]. In the current study, we showed that RIG-I/MDA5- and TBK1-directed IRF-3 phosphorylation is critical for TBEV-induced RANTES expression. In contrast, NF-kB pathway did not appear to play an essential role in stimulating RANTES transcription following TBEV infection, as evidenced by both transfection experiments with dominant-negative mutants of IkBα and pretreatment studies with NF-kB inhibitor MG132. It is conceivable that the pathways leading to activation of IRF-3 may distinct from the potential pathways that stimulate NF-kB in our system. Since ISRE promoters are activated by cellular IRFs but do not require NF-kB activation, we speculate that IRF-3-mediated activation of the ISRE is a major determinant of the induction of RANTES transcription after TBEV infection. It is important to note that other IRFs, exemplified by IRF1, have been shown to contribute to RANTES induction after respiratory syncytial virus (RSV) infection [29]. Thus, though our results support the idea that IRF3 is involved in RANTES expression during TBEV infection, the potential role for other IRFs could not be excluded. Future studies will be important and interesting to clarify whether a somewhat activated form of other IRFs could, possibly in synergy with IRF3 signaling, result in the induction of RANTES after TBEV infection.

## Conclusions

In summary, our study shows that TBEV-induced inflammatory responses in the CNS seem to be associated with a robust production of chemokines and cytokines, of which RANTES is a strong candidate for recruiting immune cells to brain tissues. In addition, our findings reveal the molecular pathway of TBEV-mediated induction of RANTES, involving activation of RIG-I/MDA5 and TBK1, with subsequent activation of IRF-3 resulting in increased RANTES expression. These findings expand the current knowledge of the role of specific chemokines during TBEV infection and render RANTES particularly interesting for further investigations in human and experimental TBE.

## Additional files

Additional file 1: Figure S1. Expression of RANTES in TBEV-infected A549 and THP-1 cells. A549 (A and B) and THP-1 cells (D and E) were inoculated with medium alone, TBEV, or UV-inactivated TBEV at an MOI of 1. Total RNA was extracted from cell lysates at 6, 24, 48, and 72 h post inoculation. RANTES mRNA was quantified by real-time PCR (A and D), and results were normalized to GAPDH and expressed as fold induction over medium alone at 6 h post inoculation. Supernatants were harvested at 6, 24, 48, and 72 h post inoculation, and levels of RANTES (pg/mL) released were determined by ELISA (B and E). Bars represent the means ± the standard deviations of three independent experiments. ^*^ *P* < 0.05 versus mock control. A549 (C) and THP-1 cells (F) were infected with TBEV at an MOI of 1. Supernatants were collected at indicated time points, and virus titers were determined by TCID_50_ assay. The results are presented as the means ± standard deviations obtained from three independent experiments. **P* < 0.05 versus mock control. NS = not significant. (TIF 372 kb) Additional file 2: Figure S2. Impact of BX795 or MG132 treatment on TBEV replication. T98G or CCF-STTG1 cells were inoculated with TBEV (MOI = 1), followed by treatment with BX795 (2 μM), MG132 (3 μM), or DMSO vehicle in the absence of serum for 36 h. Supernatants were harvested, and virus infectivity was determined by estimation of the TCID_50_ as described above. NS = not significant. (TIF 83 kb)

## Abbreviations

Ab: Antibody
AP-1: Activating protein 1
BBB: Blood-brain barrier
C/EBP: CCAAT/enhancer-binding protein
CCR: CC chemokine receptor
CNS: Central nervous system
CRE: cAMP response element
CREB: cAMP response element-binding protein
CSF: Cerebrospinal fluid
CXCL10: C-X-C motif chemokine 10
CXCL11: C-X-C motif chemokine 11
ELISA: Enzyme-linked immunosorbent assay
HE: Hematoxylin-eosin
HIV-1: Human immunodeficiency virus type 1
HSV-1: Herpes simplex virus-1
IkBα: Nuclear factor of kappa light polypeptide gene enhancer in B cells inhibitor, alpha
IKKε: Inhibitor-kB kinase epsilon
IRF-3: Interferon regulatory factor 3
ISRE: Interferon-stimulated response element
JEV: Japanese encephalitis virus
MAVS: Mitochondrial antiviral signaling protein
MCP-1: Monocyte chemoattractant protein-1
MDA5: Melanoma differentiation-associated protein 5
MHV: Mouse hepatitis virus
MOI: Multiplicity of infection
NF-IL6: Nuclear factor for interleukin 6
NF-kB: Nuclear factor kappa-light-chain-enhancer of activated B cells
PBS: Phosphate-buffered saline
PFA: Paraformaldehyde
PMA: Phorbol 12-myristate 13-acetate
PRR: Pattern recognition receptors
RABV: Rabies virus
RANTES: Regulated upon activation, normal T cell expressed, and presumably secreted
RIG-I: Retinoic acid-inducible gene 1
RSV: Respiratory syncytial virus
RT: Room temperature
SCID: Severe combined immunodeficiency
TBK1: TANK-binding kinase 1
TCID_50_: Median tissue culture infective dose
TNF: Tumor necrosis factor
TBEV: Tick-borne encephalitis virus
