# Supplementary figures and images for: Tick-borne encephalitis virus induces chemokine RANTES expression via activation of IRF-3 pathway
Source: J Neuroinflammation. 2016 Aug 30;13(1):209. doi: 10.1186/s12974-016-0665-9 (PMC5004318; doi:10.1186/s12974-016-0665-9)

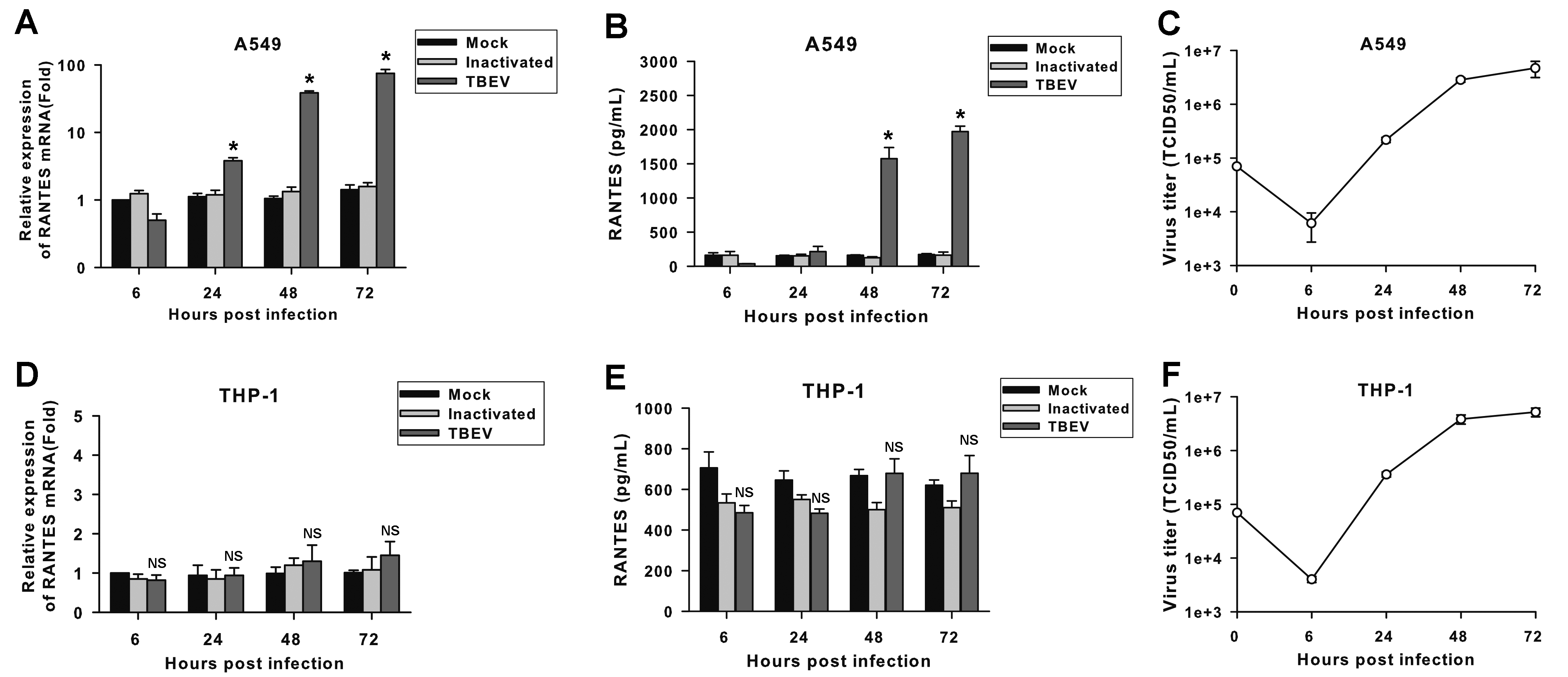

Supplement: Additional file 1: Figure S1. — Expression of RANTES in TBEV-infected A549 and THP-1 cells. A549 (A and B) and THP-1 cells (D and E) were inoculated with medium alone, TBEV, or UV-inactivated TBEV at an MOI of 1. Total RNA was extracted from cell lysates at 6, 24, 48, and 72 h post inoculation. RANTES mRNA was quantified by real-time PCR (A and D), and results were normalized to GAPDH and expressed as fold induction over medium alone at 6 h post inoculation. Supernatants were harvested at 6, 24, 48, and 72 h post inoculation, and levels of RANTES (pg/mL) released were determined by ELISA (B and E). Bars represent the means ± the standard deviations of three independent experiments. * P < 0.05 versus mock control. A549 (C) and THP-1 cells (F) were infected with TBEV at an MOI of 1. Supernatants were collected at indicated time points, and virus titers were determined by TCID50 assay. The results are presented as the means ± standard deviations obtained from three independent experiments. *P < 0.05 versus mock control. NS = not significant. (TIF 372 kb) [file 12974_2016_665_MOESM1_ESM.tif]

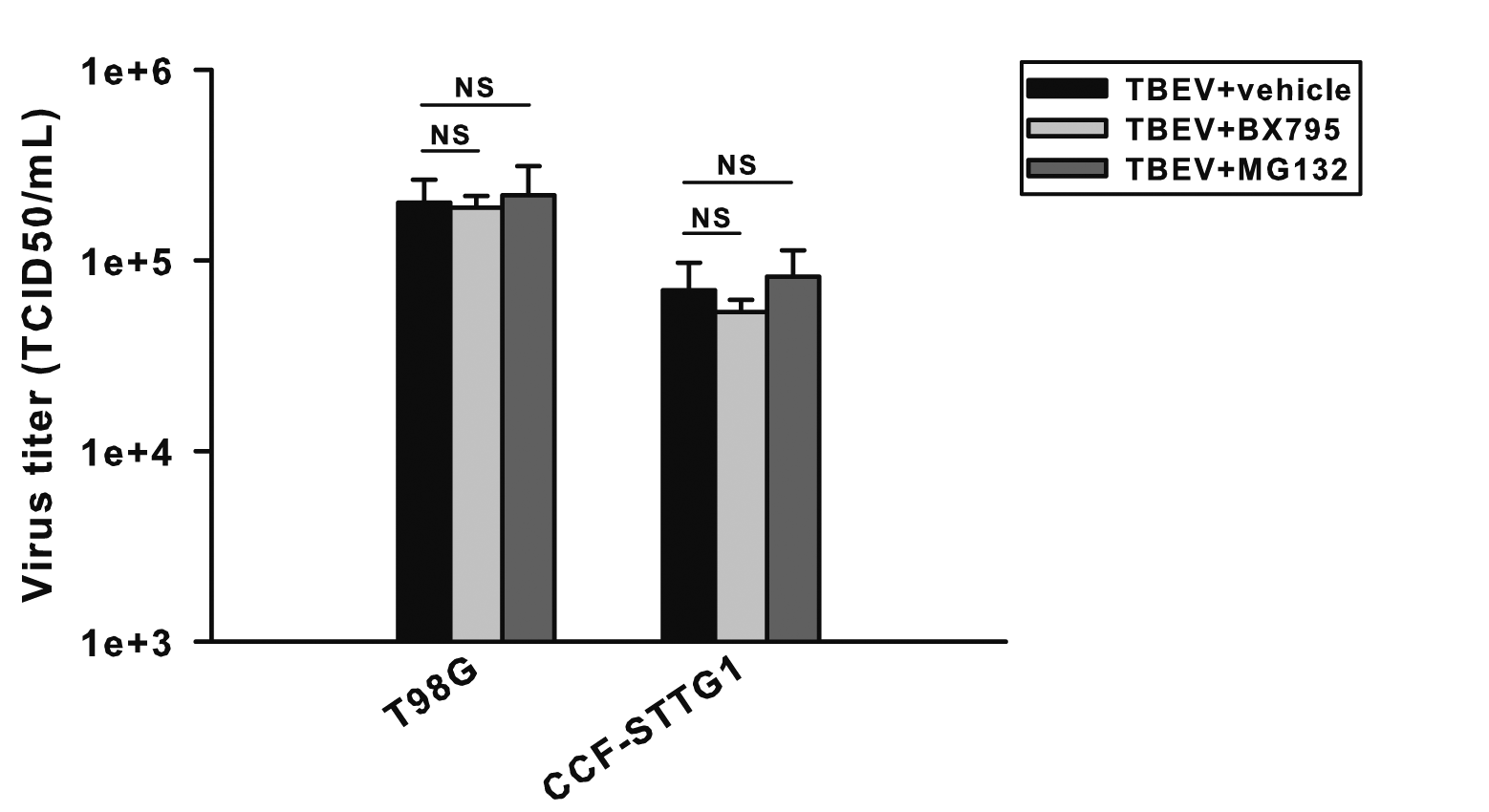

Supplement: Additional file 2: Figure S2. — Impact of BX795 or MG132 treatment on TBEV replication. T98G or CCF-STTG1 cells were inoculated with TBEV (MOI = 1), followed by treatment with BX795 (2 μM), MG132 (3 μM), or DMSO vehicle in the absence of serum for 36 h. Supernatants were harvested, and virus infectivity was determined by estimation of the TCID50 as described above. NS = not significant. (TIF 83 kb) [file 12974_2016_665_MOESM2_ESM.tif]
